# Long-term trajectories of SARS-CoV-2 neutralizing antibodies and predictive value of first dose vaccination-induced IgG-antibodies in hemodialysis patients

**DOI:** 10.1007/s11255-021-03076-2

**Published:** 2021-12-03

**Authors:** F. P. Tillmann, H. Still, Philipp von Landenberg

**Affiliations:** 1 Department of Medicine I—Nephrology, Transplantation & Medical Intensive Care, Medical Center Cologne-Merheim, University Witten/Herdecke, Ostmerheimer Str. 200, 51109 Cologne, Germany; 2 Nephrologisches Zentrum Ibbenbüren, Gravenhorsterstr. 1, 49477 Ibbenbüren, Germany; 3 LADR GmbH MVZ Nord-West, Technikerstr. 14, 48465 Schüttorf, Germany

**Keywords:** SARS-CoV-2, COVID-19, Vaccination, Neutralizing antibodies, Antibody titer trajectory, Hemodialysis, Long-term follow-up

## Abstract

**Purpose:**

The predictive value of antibody titers after the first SARS-CoV-2 vaccination and long-term trajectories of antibody titers in hemodialysis patients are unknown.

**Methods:**

SARS-CoV-2 IgG antibodies and their neutralizing effect six weeks after the first and second vaccination were analysed in 30 hemodialysis patients. IgG titers served to classify participants as responders or non-responders and to calculate sensitivity, specificity, and accuracy. Associations between potential risk factors and post-vaccine non-response were analysed by Mann–Whitney-*U* test and Chi-Squared test. Long-term follow-up analysis (ANOVA) on the evolution of neutralizing IgG-titers was performed in 24 participants 94 and 135 days after the second immunization.

**Results:**

IgG antibodies ≥ 1 AU/L (mean 9 ± 20 AU/L) after the first dose were found in 20 patients (66.7%). After the second dose only two participants (6.7%) remained sero-negative and 16.6% showed neutralizing levels below 30%, whereas 25 patients showed IgG antibodies with the high neutralizing activity of 86 ± 18%. Positive IgG antibodies 6 weeks after the first vaccination predicted vaccination effectiveness after two cycles with a specificity of 100%, sensitivity of 76%, and accuracy of 87%. Even low-dose immunosuppressive therapy increased the relative risk for non-response after the first and second dose 1.9 (95% CI 0.8–4.6) and 4.9 (95% CI 1.0–23.8) times, respectively. Over a period of about 4.5 months IgG titers slowly declined by 51% from baseline or by 0.45 AU/mL per day, respectively.

**Conclusion:**

Two cycles of SARS-CoV-2 vaccination-induced high seroconversion rates comparable to the general population. Immunosuppressive medication is a major risk factor for vaccination non-response. Mounted IgG antibodies showed a high neutralizing capacity as evidence of protective effectiveness. IgG antibodies after the first dose may serve to predict later vaccination outcome. Patients on dialysis display a more rapid decline in antibody titers on long-term follow-up compared to healthy controls.

## Introduction

So far different SARS-CoV-2 vaccines have been developed and marketed in different countries around the world. The mode of anti-viral effect of these vaccines can be described as either directly neutralizing or inducing a host immune response via purified virus components, replication-defective viral vector carrying pathogen genes, and mRNA vaccines [1]. In Germany, both gene-based and mRNA-based vaccines have been approved and are in use for several months. Patients on hemodialysis are at high risk of developing severe courses of SARS-CoV-2 infections carrying a high mortality rate [2, 3]. Despite the high risk in this vulnerable patient cohort, hemodialysis patients are vaccinated using the same vaccination scheme as in the general population with two dosages with a specified time interval between the two dosages. Observational reports have shown an insufficient immune response in end-stage-renal disease patients [4, 5] as compared to the general population with a reported efficacy of > 90% after a second dose [6]. Dialysis units are locations with a high risk of acquiring SARS-CoV-2 infections making strict hygiene protocols mandatory. Declining viral infection rates have led to calls to reduce the still strict nationwide anti-SARS-CoV-2 measures in many countries. So far, little is known about the efficacy of a first dose anti-SARS-CoV-2 vaccination to mount anti-SARS-CoV-2 IgG antibodies and if the standard two-dose fits all vaccination strategy in the general population is sufficiently effective in ESRD patients on hemodialysis. Furthermore, the neutralizing activity and the long-term decline in antibody titers have not yet been fully elucidated in this vulnerable patient cohort. Therefore, this prospective study aimed at describing the effect of SARS-CoV-2 vaccination on the long-term evolution of antibody titers and their neutralizing capacity in a cohort of hemodialysis patients.

## Methods

This is an observational, prospective single-center analysis in hemodialysis in patients > 18 years of age. Patient classified to participate in this study if they were vaccinated with a first dose of either mRNA SARS-CoV-2 vaccine (BNT162b2, Pfizer-BioNTech) or replication-defective viral vector carrying pathogen gene (ChAdOx1 nCoV-19, Oxford-AstraZeneca) at least 3 weeks prior to study inclusion. Anti-SARS-CoV-2 antibodies were re-evaluated 6–7 weeks after the second vaccination cycle. Patients with prior SARS-CoV-2 infection were not eligible to participate in this study. Post-vaccination analysis included the measurement of SARS-CoV-2 IgG-antibody titers and an evaluation of the neutralizing capacity of the IgG-antibody as described below. Patients had been vaccinated either in central vaccination facilities, by their primary care physicians or the dialysis facility itself. Dates of vaccination, type of vaccination used, and person-related data were stored centrally in a password-protected data sheet.

Past medical history of COVID-19 and outcomes before the start of the study were determined by the medical staff of the facility in all participants prior to the start of the study. Demographic data (age, sex, dialysis vintage, BMI, prior history of transplantation, online conductivity Kt/V clearance [OCM-device™ Fa. Fresenius Medical Care], estimated glomerular filtration rate according to the CKD-EPI formula in ml/min/1.73 m^2^ BSA, albumin in mg/dl, type of hemodialysis access [fistula, graft, catheter], candidacy for renal transplantation, active immunosuppressive medication at the time of antibody titer evaluation, diabetes mellitus, active malignancy, active hepatitis, and anti-HBs-titers, CRP levels in mg/dl, parathyroid hormone levels in pmol/l, calcidiol levels in nmol/l, and calcitriol levels in pg/ml) were recorded in every patient at baseline. Major outcome variables were IgG-antibody titers categorized in negative or positive (≥ 1.0 AU/ml), and the neutralizing capacity of positive antibody titers in % (0−< 30% up to 100%).

The study was approved by the local ethics committee “*Ethikkommission der Ärztekammer Westfalen-Lippe und der Westfälischen Wilhelms Universität*” in Münster (2021–131-f-S) and conducted in line with the Declaration of Helsinki and the European Union Clinical Trials Directive 2001/20/EC (EU CTD). Written informed consent to participate and to publish was obtained from all individual participants included in the study. All patients gave informed consent prior to study participation.

## Laboratory measurements of SARS-CoV-2 antibodies

### SARS-CoV-2 IgG antibody test assay

In this study, a commercially available immunoassay was used for antibody detection, the anti-SARS-CoV-2 S-RBD IgG (Snibe Diagnostics, New Industries Biomedical Engineering Co., Ltd [Snibe], Shenzhen, China). SARS-CoV-2 S-RBD IgG is a chemiluminescent immunoassay (CLIA) that determines IgG Ab against the RBD of the Spike (S) protein of the virus, in human serum or plasma. All analyses were performed on MAGLUMI™ 4000 instrument (Snibe Diagnostics), with results expressed in AU/L. The assay has a clinical sensitivity between 74.5% (days post-onset of Symptoms 0–7) and 100.0% (days post-onset of Symptoms > 15), and a specificity of 99.6% [95% confidence interval (95% CI) 98.7%–100.0%]. Results were reported in AU/L from 0 to 100. Values greater 100 were reported > 100 AU/L. For analysis, these data were categorized into three classes of IgG-levels of 0, 1–100, and > 100 AU/L. To increase comparability between different investigations data antibody titers in AU/mL can be multiplied by 4.3 to receive values in BAU/mL. *Definition of seroconversion or responder status after the second dose: *

A value of ≥ 30 AU/ml about 6 weeks after the second vaccination cycle was considered “seroconversion or responder status” as opposed to “missing or incomplete seroconversion or nonresponder status”.

### SARS-CoV-2 IgG neutralizing test assay

We used the ELISA-based GenScript SARS-CoV-2 Surrogate Virus Neutralization Test Kit (GenScript 105 Biotech, Piscataway Township, USA). The test was used according to the manufacturer's recommendations. Samples were diluted in sample buffer and incubated at 37° for 30 min in the 96-well microtiter plates provided, followed by the respective wash and incubation cycles, including controls, and required reagents. The microtiter plates are coated with the “host cell receptor” angiotensin-converting enzyme 2 (ACE2). Samples containing SARS-CoV-2 neutralizing antibodies block the protein–protein reaction between ACE2 and the added (S)-RBD-horseradish peroxidase conjugate. The reduced color change upon the addition of chromogenic substrate can be measured photometrically. Optical density (OD) was measured at 450 nm using the microplate reader of a VIRCLIA® automation system. The signal to cut-off ratio was calculated and the values printed and interpreted according to the manufacturer's protocol and results were reported in %. *Definition of neutralizing capacity of SARS-CoV-2 IgG levels after the second dose:* A value of ≥ 30% about six weeks after the second vaccination cycle was considered “fully neutralizing capacity” as opposed to “missing or incomplete neutralizing capacity”.

### Statistical analyses

Data are shown as mean plus-minus standard deviation (SD) or percentage, according to the type of variable analysed. We used the Chi-Squared test for associations between qualitative variables and the Mann–Whitney-*U* test for quantitative variables. One-way ANOVA with post-hoc Bonferroni adjustment for multiple comparisons was applied to test for differences in SARS-CoV-2 IgG-titers over long-term follow-up. Values of *p* < 0.05 were considered statistically significant. Statistical analyses were performed using SPSS, IBM Corp., Armonk, NY.

## Results

### Patient characteristics

In total 30 patients, 22 men (73.3%) and 8 women (26.7%), were included in this analysis. Three patients (10.0%) were anti-HBcore-antibody positive and only one patient suffered from active malignancy of the prostate. Four patients had a failing renal transplant in situ. Seven patients were dialyzed via a central dialysis catheter. Seven patients (23.3%) received active but low-dose immunomodulatory therapy in form of steroids alone (*n* = 2), a combination of steroids and calcineurin-inhibitors (*n* = 4) or a combination of steroids and cyclophosphamide (*n* = 1). No patient received mycophenolate or its derivatives. SARS-CoV-2 vaccination was performed in 28 (93.3%) patients via mRNA-based and only two via vector-based vaccines. All except two patients were dialyzed via polysulfone membranes. Further cohort characteristics are shown in Table 1.

**Table 1:** Basic cohort characteristics

Cohort of 30 hemodialysis patients			
Male sex (%)	73.3	Kt/V	1.2 ± 0.4
Vaccine type (%)	mRNA 90 Vector 10	eGFR	8 ± 5
Prior KTx (%)	13.3	Albumin g/L	3566 ± 618
HD-catheter (%)	23.3	Age in years	62.3 ± 15.3
KTx waitlisted (%)	46.7	Days 1te to 2te vaccination	44 ± 17
IS-therapy (%)	23.3	HD-vintage in years	3.82 ± 3.55
Diabetes (%)	26.7	CRP mg/dL	0.6 ± 0.8
Active malignancy	3.3	PTH pmol/L	36 ± 37
Positive anti-HBs-titer (%)	46.7	Calcidiol nmol/L	72 ± 21
BMI	26.0 ± 5.3	Calcitriol pg/mL	17 ± 7

HD = hemodialysis, KTx = kidney transplantation, IS = immunosuppressive, eGFR = CKD-EPI formula in ml/min/1.73 m^2^ BSA

### SARS-CoV-2 IgG antibody titers and neutralizing capacity after the first dose

About 6–7 weeks after application of the first dose the mean SARS-CoV-2 IgG antibody titer was 9 ± 20 AU/L, whereas 10 (33.3%) patients had no detectable antibody titers. Only six patients (20%) showed neutralizing levels over 30% (mean 50 ± 20%), whereas 24 patients showed no measurable neutralizing activity. Despite the relatively low titer 20 patients (66.7%) were already able to mount antibodies despite only on the cycle of vaccination. Without exception patients with a positive IgG titer after the first dose were all able to mount a full antibody response after the second immunization cycle.

### SARS-CoV-2 IgG antibody titers and neutralizing capacity after the second dose

About 6–7 weeks after application of the second dose 21 (70.0%) patients had antibody titers > 100 AU/L, seven patients (23.3%) showed titers between 1 and 100 AU/L, and only two patients (6.7%) had no detectable antibodies. Only five patients (16.6%) showed neutralizing levels below 30%, whereas 25 patients showed IgG antibodies with the high neutralizing activity of 86 ± 18%. The 20 patients who were able to mount an antibody response already after the first vaccination cycle showed an even higher neutralizing effect of 91 ± 17% after the second dose.

### Characteristics of incomplete and/or non-responders after the first dose

Contrary to the definition of missing or incomplete response after the second dose, non-response after the first dose was defined as 0 or non-detectable IgG antibodies prior to application of the second vaccination. 20 patients showed positive IgG antibody titers, whereas in ten patients no sero-reactivity was detectable after the first dose. Analysis of continuous (Mann–Whitney-*U* test) variables did not show any difference between both regarding the following variables: BMI, age, Kt/V, eGFR, dialysis vintage, albumin, c-reactive protein, parathyroid hormone, calcidiol, and calcitriol levels. Patients on immunomodulatory therapy showed a 1.9 (95% CI 0.8–4.6) times higher relative risk of “non-response status” to vaccination as compared to patients without. Analysis of categorical variables was not possible due to cell-numbers ≥ 5.

### Characteristics of incomplete and/or non-responders after the second dose

In sum, five patients (16.6%, three men and two women) were classified as “missing or incomplete seroconversion or non-responder status”. The same patients were also classified as “missing or incomplete neutralizing capacity status”. Nevertheless, only two patients showed no detectable antibody response after full vaccination at all. Of note, three of these patients were on an immunosuppressive regimen consisting of steroids and calcineurin-inhibitors. Non-parametric testing (Mann–Whitney *U* test) only pointed towards a possible difference in dialysis vintage between responders and non-responders (4.31 ± 3.66 vs. 1.37 ± 1.39 years, *p* = 0.037), whereas no signal was found for BMI, age, Kt/V, eGFR, albumin, c-reactive protein, parathyroid hormone, calcidiol, and calcitriol levels. Patients on immunomodulatory therapy showed a 4.9 (95% CI 1.0–23.8) times higher relative risk of “incomplete or non-response status” to vaccination as compared to patients without. Analysis of categorical variables was not possible due to cell-numbers ≥ 5.

### Predictive value of IgG-antibody positivity on vaccination effectiveness after two dosages

In a further step, we tested the performance characteristics of a determination of SARS-CoV-2 IgG antibody measurement 6–7 weeks after the first vaccination cycle regarding vaccine efficacy 6–7 weeks after completion of the full vaccination schedule. Under the assumption of a positive test result in cases of IgG antibody > 1 AU/L and positive outcome result in cases of IgG levels > 30 AU/L we obtained the following results: sensitivity 76%, specificity 100%, false-positive-rate 0%, false-negative-rate 55%, and accuracy 87%.

### Long-term evolution of IgG-antibodies and their effectiveness after two dosages

**Fig. 1:**
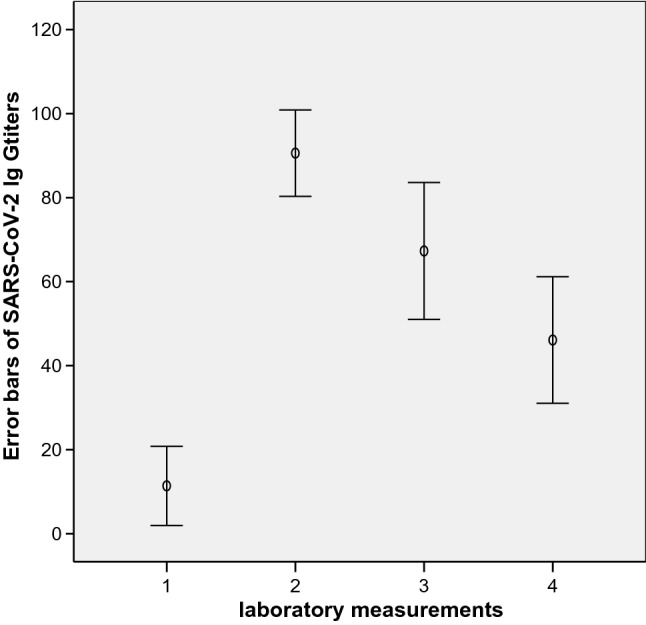
Error bars of SARS-CoV-2 IgG antibodies showing the means and respective 95% CI over 4 measurement cycles on long-term follow-up in 24 hemodialysis patients, one way ANOVA *p* <  = 0.001 with the exceptions of comparisons between the second and third measurements (*p* = 0.065) and between the third and fourth measurement (*p* = 0.120)

From the initial 30 participants of the cohort 24 patients were further analysed with respect to long-term evolution of IgG-antibody titers and their neutralizing capacity after two standard dose vaccinations. Six patients could not be evaluated due to the following reasons: one patient died due to acute pancreatitis, two patients received a booster vaccination in the meantime, and three patients were classified as non-responders. The evolution of IgG-antibodies in AU/mL and their neutralizing capacities are shown in Table 2. One way ANOVA with post-hoc Bonferroni adjustment of IgG-antibody titers showed significant differences in antibody titers (*p* ≤ 0.001) with the exceptions of comparisons between the second and third laboratory measurements, and between the third and fourth evaluation (respective error bars are shown in Fig. 1). Declining rates of antibody titers between the laboratory measurements are shown in Table 3.

**Table 2:** Long-term evolution of SARS-CoV-2 IgG antibodies in AU/mL and the respective neutralizing effect in 24 patients on hemodialysis

Long-term follow-up data in 24 hemodialysis patients					
		1	2	3	4
IgG	Mean	11	91	67	46
	STD	22	24	29	36
	95% CI	2–21	80–101	51–84	31–61
cPASS	Mean	25	83	69	65
	STD	20	26	30	25
	95% CI	17–34	72–94	57–82	54–76
Days	Mean	44	35	94	135
	STD	19	19	32	36
	95% CI	35–52	27–43	80–108	120–150

Measurement 1 was performed on average 11 days after the first vaccination, measurements 2–4 were performed on average 35, 94, and 135 days after the second standard dose vaccination

**Table 3:** Decline in antibody titers between the second, third and fourth laboratory measurements in 24 hemodialysis patients in % and AU/mL per day

Decline in SARS-CoV IgG antibody titers		
	% from baseline	AU/mL/day
2 to 3	74	− 0.41
3 to 4	69	− 0.56
2 to 4	51	− 0.45

Values six weeks after the second vaccination were taken as baseline value

## Discussion

This study aimed to investigate the effectiveness of the first SARS-CoV-2 vaccination cycle on the evolution of antivirus antibodies and their neutralizing capacity in hemodialysis patients. Our results are in line with a very high effectiveness of mRNA- and vector-based vaccines as reported in the general population. In our cohort of hemodialysis patients only 6.7% were classified as non-responders without any antibody formation and only 9.9% showed a reduced evolution of neutralizing antibodies between 1 and 30 AU/mL. Our response rates after the second vaccine dose are in line with previous results in ESRD patients on hemodialysis e.g., Anand and co-workers reported a reduced effectiveness in 26.5% of participants 28 days after the second dose in 355 hemodialysis patients [7]. Other investigations found response rates of over 80% [8] or over 90% [9–11]. These data are comparable or even superior to antibody response rates in symptomatic hemodialysis patients after infection with the wild-type virus [12]. Interestingly, serial antibody measurements in 122 hemodialysis patients after infection showed raising titers with a peak at four months [13]. Therefore, the timing of antibody titer measurement might be a relevant potential source of discrepancies between investigations partly explaining differences in rates of seroconversion also after vaccination. Our study adds to these reports so far, as we could not only confirm the presence of SARS-CoV-2 antibodies in 93.3% of our participants but also their neutralizing capacity with a mean value of 86% after two vaccination cycles.

In contrast to the growing number of studies on vaccine efficacy after a full vaccination with two dosages, the data on the effect after a first vaccination in hemodialysis patients is still scarce. The first report on first-dose effects in hemodialysis patients was reported by Torregiani and co-workers in April this year [14]. They reported a mean antibody titer of 8 U/ml comparable to our findings of 9 AU/mL but found only a reduced responder rate of 35.6% as opposed to our rate of 66.6%. A reason for this difference might be the reduced time-interval of three weeks between application of the first dose and antibody measurement in their study and 6–7 weeks in our investigation. Other investigations reported sero-conversion rates of 79.8% four weeks after the first dose of BNT162b2 mRNA vaccine in 94 hemodialysis patients [15] or 42% in 50 patients in a study by Zitt and co-workers [16]. Of note, the authors of the latter applied a different definition of sero-conversion using the assay-specific cut-off value of 33.8 binding activity units per milliliter four weeks after the first dose [16]. Therefore, in interpreting and comparing study results not only the timing of antibody determination with respect to vaccination but also different definitions of response and non-response should be considered. Our definition of a positive response of an antibody titer > 0 AU/mL after the first dose of vaccination showed promising performance parameters with a specificity of 100% and an accuracy of 87%. Immunosuppressive therapy even at low doses increase to risk for non-response to vaccination in our cohort to 1.9 and 4.9 times after the first and second vaccination dose, respectively. Therefore, in our view, the purpose of checking the effect after only one dose could be to offer non-responding vaccine recipients a modified vaccination schedule or a reduction in any immunosuppressive therapy already at this early stage. The slight statistical association between fewer years on hemodialysis and lower vaccine response is, in our opinion, more likely since patients with recent graft failure some months ago were still on low-dose immunosuppression. Finally, we could not detect an association between antibody response and vitamin D levels or success to hepatitis B vaccination.

Major limitations of our investigation are the reduced patient numbers and the single-center observational non-randomized design of our study protocol. Furthermore, severe selection bias towards patients interested in participating in this investigation might have occurred.

As opposed to one report of hemodialysis patients after wild virus infection [13], we could demonstrate a continuous fall in antibody titers over a period of 4.5 months after the second vaccination dose. Recently, long-term data on titer progression have also been published. For example, Levin and co-workers were able to describe the IgG titer trajectories of neutralizing SARS-CoV-2 antibodies over six months in a large cohort of employees of a health facility [17]. Interestingly, their data are comparable to the results of this investigation in a greater and more rapid decline in total IgG-titers as compared to neutralizing antibodies. Another investigation reported an estimated anti-spike IgG half-life of 184 days in a representative population-based cohort of over 7.000 participants in the United Kingdom [18]. Another analysis reported an even more pronounced but expected fall in IgG titers comparable to titers after the first vaccination dose in 122 healthy volunteers after six months [19]. Generally, this fall is linked to the fact, that vaccination does normally only induce short-lived plasma cells as compared to log-lived plasma cells after wild virus infections. Most recently, Speer and co-workers analysed antibody trajectories in 124 hemodialysis and 41 peritoneal dialysis patients [20]. This prospective multi-center study found a decline in IgG antibody titers after 12 weeks of 55% in participants on hemodialysis and 45% in patients on peritoneal dialysis. Therefore, these data combined with our findings of an IgG antibody titer half-life of about 135 days are suggestive of a more rapid decline in antibody titers in hemodialysis patients. Despite reports on mild clinical courses of wild-type infections after vaccination [21], no conclusive information on the effect of falling titers on break-through infections in hemodialysis patients have yet been published. Consequently, repeat antibody testing and booster vaccinations seem to be a sensible strategy in ESRD patients given the high vulnerability of this patient population to dismal clinical courses or even death after SARS-CoV-2 infection.

In conclusion, our results confirmed high antibody titer conversion rates after two full cycles of SARS-CoV-2 vaccination and showed the neutralizing capacity of the mounted antibodies. Even low-dose immunosuppressants seem to impair a protective antibody response in hemodialysis patients. Hemodialysis patients seem to be prone to a more rapid decline in antibody titers as compared to healthy cohorts. Continuous antibody surveillance and probably booster vaccinations might serve in the near future to protect these patients from unfavorable clinical courses after SARS.coV-2 contact or break-through infections.
